# Optimized N application improves N absorption, population dynamics, and ear fruiting traits of wheat

**DOI:** 10.3389/fpls.2023.1199168

**Published:** 2023-08-29

**Authors:** Xiangqian Zhang, Yunji Xu, Shizhou Du, Yuqiang Qiao, Chengfu Cao, Huan Chen

**Affiliations:** ^1^ Crops Research Institute, Anhui Academy of Agricultural Sciences, Hefei, Anhui, China; ^2^ Joint International Research Laboratory of Agriculture and Agri-product Safety, The Ministry of Education of China, Yangzhou University, Yangzhou, Jiangsu, China

**Keywords:** N use efficiency, leaf area, light interception rate, ear fruiting traits, yield

## Abstract

Optimizing the N application amount and topdressing ratio can increase crop yield and decrease N loss, but its internal mechanisms have not been well studied, especially from the aspects of population dynamics and structure, ear fruiting traits. Here, field experiments, with three N rates 120 (N1), 180 (N2), and 240 (N3) kg N ha^-1^ and three N topdressing ratios T1 (7:3), T2 (6:4), and T3 (5:5) were conducted. At the same N level, results showed that the N accumulation amounts in the leaf, grain, and plant in T2 were higher than in T3 and T1, and increasing 60 kg N ha^-1^ (N3 compared to N2, N2 compared to N1) significantly enhanced N accumulation amounts. The effect of the N topdressing ratio on partial factor productivity of applied N was consistently T2 > T3 > T1, but T1 was more conducive to improving N utilization efficiency for grain and biomass production. After the jointing stage, compared to T1 and T3, T2 was more conducive to increasing the population growth rate of plant height, leaf area index, leaf area growth rate, dry matter weight, dry matter accumulation rate, light interception rate, and spikelets of population, and the above-mentioned indexes of population could be significantly enhanced by increasing 120 kg N ha^-1^. T2 increased the fruiting spikelets per ear, grains per ear, grain weight per ear, fruiting rate per ear, grain filling rate per ear, and yield but decreased the sterile spikelets at the top and bottom and imperfect grains per ear. Increasing N from 120 kg ha^-1^ to 180 kg ha^-1^ or from 180 kg ha^-1^ to 240 kg ha^-1^ significantly enhanced yield. The N accumulation amount in the grain, leaf, plant, leaf area growth rate, dry matter accumulation rate, light interception rate, population spikelets, fruiting spikelets per ear, grain filling rate, and yield were significantly positively correlated with each other. This study demonstrates a suitable N application rate with a N topdressing ratio 6:4 would more effectively improve N efficiency, population dynamics, structure, ear fruiting traits, and yield, but the effect of the N topdressing ratio is not as significant as that of increasing 60 kg N ha^-1^.

## 1 Introduction

Wheat is one of the most important crops in the world, with figures showing a harvested area of 214.3 million ha and a global production of 734 million tons in 2018 (Illescas et al., 2020). As an essential nutrient for crop growth and development, nitrogen (N) is a major determinant of grain yield in wheat cropping systems, increasing the N application rate has contributed significantly to the increase of wheat yield in the world (Zörb et al., 2018). However, the rates of crop yield growth have slowed down in the past 20 years and have even become stagnated in some countries and regions despite enhanced N fertilizer input (Grassini et al., 2013; Che et al., 2015). The N fertilizer use efficiency in wheat production is abysmally low, i.e., around 30–35%, implying that only 30–35% of applied N is absorbed and utilized while the remaining 65–70% is lost into the environment (Sinha et al., 2020). The high rate and unsuitable timing of N application are major problems in wheat production. This has resulted in low N use efficiency, low yield and economic benefit, and high environmental costs (Lu et al., 2015).

Given the key indexes of yield formation and N application rate are not always positively correlated, to achieve high yield and N use efficiency many useful N application techniques have been developed and researched, and these N application technologies mainly focus on optimizing the total N input, adjusting the topdressing time, and decreasing the N losses. Ravier et al. (2017) found that decreasing the N application rate at the seedling stage had no impact on yield, grain protein content, and dry matter weight but increased N uptake rate and NUE (N use efficiency), as wheat has a strong low-N tolerance at the seedling stage. Sui et al. (2013) observed that optimizing N topdressing strategies enhanced rice yield by 8.2–12.6% and NUE by 57.14–138.3% when compared to local farmers’ fertilization practices. Likewise, others also successfully improved crop yield, NUE, and grain quality by optimizing the N application amount and topdressing ratio (Qu et al., 2018; Liu et al., 2019; Sun et al., 2020). Therefore, in terms of optimizing N rates and its splitting during the crop growth cycle, split application of N, where some N is applied before sowing and some is applied at later growth periods, contributes to synchronize N supply with crop N demand and reduce N loss. However, an unsuitable N application rate and time will usually cause a lack of N in a certain crop growth periods, and the N deficiency has been proved to make leaf area, biomass accumulation, plant N concentration, plant photosynthetic capacity, and crop growth rate decrease, which are strongly associated with the grain filling rate, yield, and harvest index (Liu et al., 2019; Dong et al., 2022).

Increasing N rates is unlikely to be effective in increasing crop yields; excessive use of N fertilizers and inappropriate application methods will cause excessive amounts of N in the soil since it cannot be absorbed by crops, limiting instead plant growth and reducing N use efficiency due to the asynchrony between N supply and N requirements (Ercoli et al., 2013; Liu et al., 2019);. Better management and appropriate use of N fertilizers are a convenient and effective way to improve crop growth, population structure, N accumulation, N use efficiency, and yield components (Peng et al., 2010; Ciampitti and Vyn, 2012). Although a suitable N application amount and topdressing ratio can obviously increase wheat yield (Shi et al., 2007; Zhao and Si, 2015), the internal mechanisms of yield formation or increase have not been well studied, especially from the aspects of population dynamics and structure, ear fruiting traits, N absorption and utilization, and the potential relationships of mutual inhibition and promotion among them. Therefore, a relevant field experiment was conducted (i) to clarify the variation trend of N accumulation, N use efficiency, population dynamics and structure, ear fruiting traits and wheat yield, which respond to different N application amounts and N topdressing ratios; (ii) to reveal the suitable N application amount and topdressing ratio with which N efficiency, population dynamics, and yield are effectively improved so as to maximize the yield and N benefits; and (iii) to identify the potential relationships among the above-mentioned key indexes of yield formation.

## 2 Materials and methods

### 2.1 Site description

Field experiments were conducted in a Baihu farm (31°53’ N, 117°14’ E; 29.8 m a.s.l.), Lujiang County, Anhui Province in China from November 2019 to May 2021 (the climate being basically the same over the 3 years). The 3-year experimental site (in the same location) is located in the southeast of the country with an annual single rice-wheat rotation system. The region is classified as having a subtropical monsoon climate. The annual mean temperature (2019∼2021) is 16.0°C, accumulated temperatures above 10°C are about 5100°C, and annual mean precipitation is about 1150 mm. Soil samples were collected from the research field at a depth of 0–20 cm for analyzing soil properties before wheat sowing and basal fertilizers application. The soil’s physical and chemical properties at depths of 0–20 cm at the beginning of the experiment were as follows: available N 98.09 mg kg^-1^, available P 7.46 mg kg^-1^, available K 93.8 mg kg^-1^, total N 1.19 g kg^-1^, total P 0.61 g kg^-1^, organic matter 18.31 g kg^-1^, and pH 5.8.

### 2.2 Experimental design

The field experiment was laid out in a two-factor completely randomized design with three replicates for each treatment. One factor was the N application level, consisting of three N levels, i.e., 120, 180, and 240 kg ha^−1^ (referred as N1, N2, and N3, and the highest N application rate in this study was slightly lower than the conventional N application rate in the test area). Another factor was the ratio of basal N to topdressing, including three N topdressing ratios, i.e., 7:3, 6:4, and 5:5 (referred to as T1, T2, and T3). Therefore, nine treatments were established, namely N1T1, N1T2, N1T3, N2T1, N2T2, N2T3, N3T1, N3T2, and N3T3. For all plots, 120 kg P ha^-1^ and 120 kg K ha^-1^ were applied as basal fertilizer before sowing, and the topdressing N was applied at the jointing stage of wheat, N was applied as urea (46.4% N), and P and K were supplied as calcium superphosphate (12% P_2_O_5_) and potassium chloride (60% K_2_O), respectively. The plot size was 3 m × 4 m with a row space of 25 cm, and the distance between neighboring plots was 50 cm. A local wheat variety of “Ningmai 13” (widely planted in the region) was selected and planted on 6 November with a density 300 × 10^4^ ha^-1^ for basic seedlings and was harvested on 24 May of the following year.

### 2.3 Sampling and measurements

N accumulation: at the maturity stage, 1-m rows of consecutive plants in each experimental plot were clipped at soil surface, and separated into three fractions: leaves, stems plus petioles, and grains. The plant samples were oven-dried at 80°C until reaching a constant weight, and then we calculated aboveground dry matter (ADM) weight at the maturity stage. Each ADM sample (leaves, stems plus petioles, and grains) was ground separately using 1mm of screen mesh. Total N content in the above-ground parts were determined using the semi-micro Kjeldahl method (FOSS-2300, FOSS Analytical A/S, Denmark) where total N accumulation = dry matter weight of tissues × total N content (Shi and Yan, 2013).

N utilization efficiency: partial factor productivity of applied N (PFP) = kg grain yield with N application/kg N applied); N utilization efficiency for grain production (kg kg^-1^) = grain yield/total N content in the above-ground plant; N utilization efficiency for biomass production (kg kg^-1^) = dry matter weight of population at maturity stage/total N content in the above-ground plant.

Dynamic changes of plant population height: the average height of wheat population was measured at jointing and flowering stages, the height of wheat plants was the distance from soil surface to the top of leaves, and the population growth rate of plant height = average plant height (mm)/number of days during the measurement of two growth periods (d).

Dynamic changes of population leaf area: leaf area was measured using a portable leaf area meter (Model Li-3000C, Li-cor, Lincoln, Nebraska, USA), and then LAI (leaf area index) was calculated as leaf area per unit land area. In total, 20 wheat plants in each treatment were sampled and measured at the jointing, flowering, and middle of filling stages in 2020 and 2021. Leaf area growth rate = population leaf area (m^2^)/number of days during the measurement of two growth periods (d).

Dynamic changes of dry matter accumulation of population: to obtain the dry matter weight, 20 wheat plants in each plot were sampled (at the jointing, flowering, and maturity stages) and oven dried at 80°C for 72 h till they reached a constant weight. The dry matter accumulation rate of population = dry matter weight of population (kg)/number of days during the measurement of two growth periods (d).

Dynamic changes of the light interception rate of the population: photosynthetically active radiation (PAR) was measured by a SUNSCAN Canopy Analysis System (Delta company, Britain) at the booting, flowering, initial of filling, and end of filing stages of wheat in 2020 and 2021, respectively. The PAR was calculated according to the difference value between the PAR of the top and bottom of the wheat population, and the measurements were performed at 9:30–12:00 h, Beijing standard time, on sunny days. The measurement locations were chosen randomly, and 10 replications were performed for each plot. LI (light interception rate) was calculated using the following formula (Xue et al., 2015; Tsujimoto et al., 2017): LI = (1-Io/lt)×100%, where LI is the percentage of light that is intercepted, and Io and It are the PAR of wheat population at top and bottom, respectively.

Spikelet number of population, fruiting spikelets per ear, sterile spikelets, grains per ear, grain weight per ear, imperfect grains, and fruiting rate per ear were determined at harvesting for an area of 2 square meters for each plot in 2020 and 2021. The percentage of fruiting spikelets was calculated by dividing the number of fruiting spikelets based on the number of total spikelets panicle^-1^.

### 2.4 Data analysis

All data was expressed as means over three replicates. ANOVA was conducted by using SPSS 21.0 software with the general linear model-univariate procedure (IBM, Armonk, New York, USA). A one-way analysis of variance (ANOVA) was performed on each measurement index in the paper to compare differences among N level and N topdressing treatments for each year. All treatment means were compared for any significant differences by the Duncan’s multiple range tests at the 5%level using the SPSS 21.0 software package for Windows.

## 3 Result

### 3.1 Effects of optimizing N application on N absorption and utilization

#### 3.1.1 Plant N uptake and accumulation

**Figure 1:**
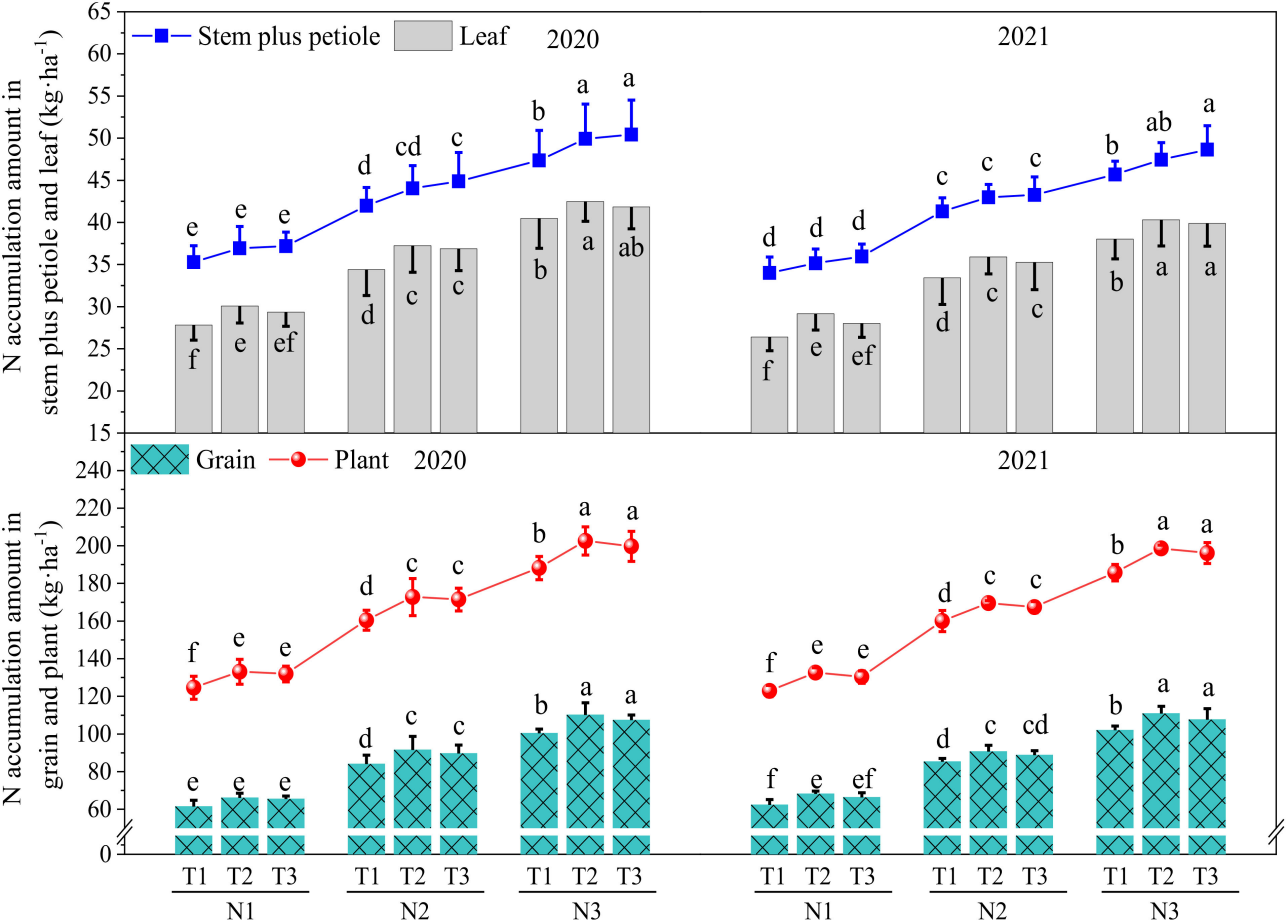
The Effect of optimizing N application on plant N uptake and accumulation at maturity stage. Values were means ± SD. Different letters indicate a significant difference within the same year under the treatments of three N levels and three N topdressing ratios by Duncan test (ANOVA) at the 5% level. Three N fertilization levels: N1, 120 kg ha^-1^; N2, 180 kg ha^-1^; and N3, 240 kg ha^-1^. Three N topdressing ratios: T1, 7:3; T2, 6:4; and T3, 5:5.

At (**Figure 1**) the same N level (N1, N2, or N3), the total amount of N accumulation in the stem plus petiole in T3 was higher than that in T1 and T2, and the differences between T3 and T2 were insignificant, while the differences between T3 and T1 were significant at N2 and N3 in 2020. The effect of the N topdressing ratio on the amount of N accumulation in leaf, grain, and plant were consistently T2 > T3 > T1, and the differences between T2 and T1 were significant (except for grain at N1 in 2020), while the differences between T2 and T3 were insignificant. With the same N topdressing ratio (T1, T2, or T3), the effect of N application amount on N accumulation in the stem plus petiole, leaf, grain, and plant were consistently N3 > N2 > N1, and the differences between N2 and N1, N3, and N2 were significant, indicating that increasing 60 kg N ha^-1^ (N3 compared to N2, N2 compared to N1) significantly enhanced N accumulation amount in wheat. In addition, we also found that the amount of N accumulation at maturity stage were consistently plant > grain > stem plus petiole > leaf.

#### 3.1.2 N utilization efficiency

**Figure 2:**
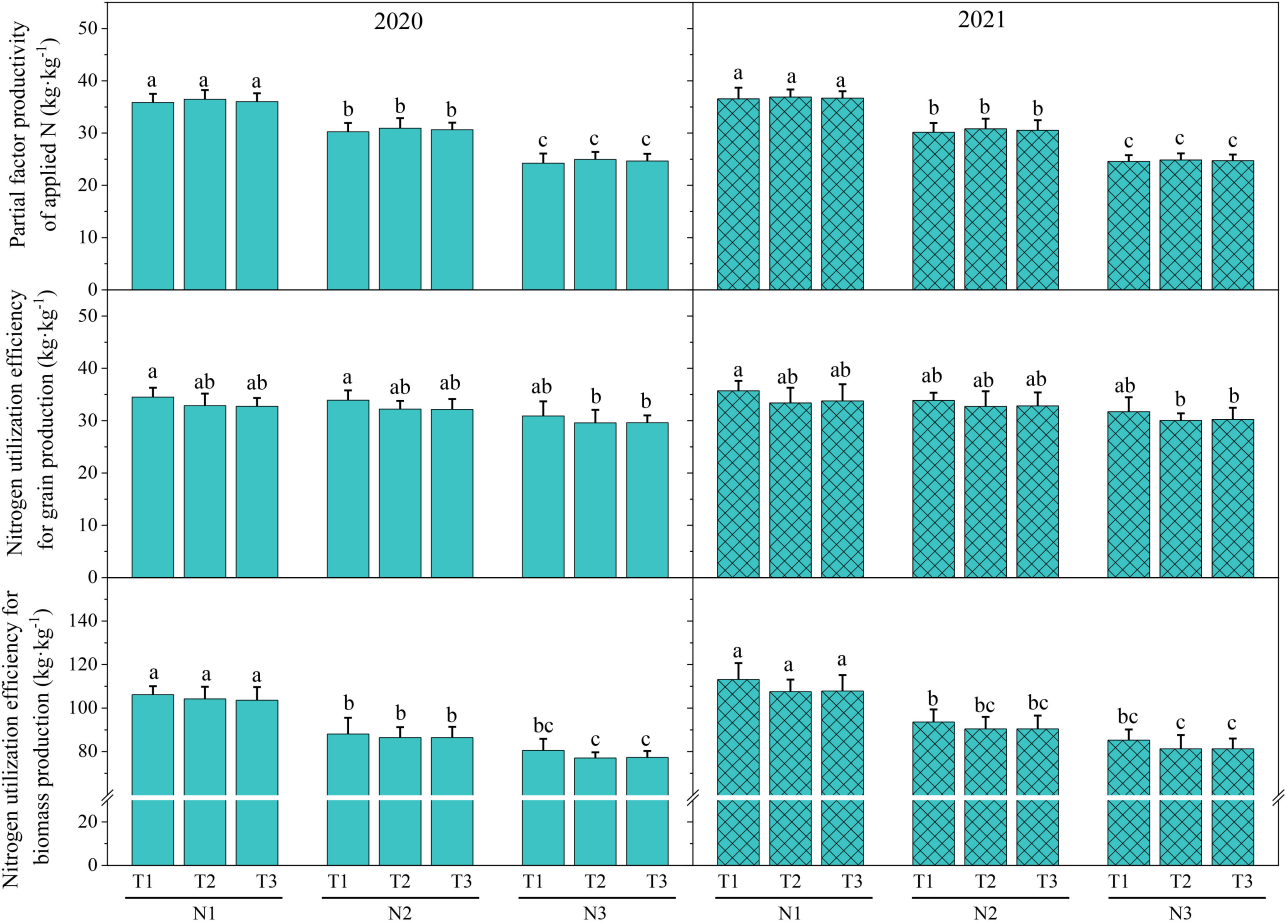
The Effect of optimizing N application on N use efficiency. Values were means ± SD. Different letters indicated significant difference within the same year under the treatments of three N levels and three N topdressing ratios by Duncan test (ANOVA) at the 5% level. Three N fertilization levels: N1, 120 kg ha^-1^; N2, 180 kg ha^-1^; and N3, 240 kg ha^-1^. Three N topdressing ratios: T1, 7:3; T2, 6:4; and T3, 5:5.

As shown in **Figure 2**, at the same N level (N1, N2, or N3), the effect of the N topdressing ratio on partial factor productivity of applied N was consistently T2 > T3 > T1 in 2020 and 2021, and the values of N utilization efficiency for grain production and N utilization efficiency for biomass production were the highest in T1. However, at the same N level, the differences in partial factor productivity of applied N, N utilization efficiency for grain production, and N utilization efficiency for biomass production among T1, T2, and T3 were insignificant. With the same N topdressing ratio (T1, T2, or T3), the effect of N application amount on partial factor productivity of applied N, N utilization efficiency for grain production, and N utilization efficiency for biomass production were consistently N1 > N2 > N3. The partial factor productivity of applied N in N1 were significantly higher than in N2 and in N2 significantly higher than that in N3, indicating that increasing 60 kg N ha^-1^ significantly decreased partial factor productivity of applied N. Compared to N3, N1 for T1, T2, and T3 significantly decreased N utilization efficiency for biomass production in 2020 and 2021, indicating that increasing 120 kg N ha^-1^ would significantly decrease N utilization efficiency for biomass production. With the same N topdressing ratio, the differences in N utilization efficiency for grain production among N1, N2, and N3 were insignificant.

### 3.2 Effects of optimizing N application on population dynamics

#### 3.2.1 Dynamic changes of plant population height

**Figure 3:**
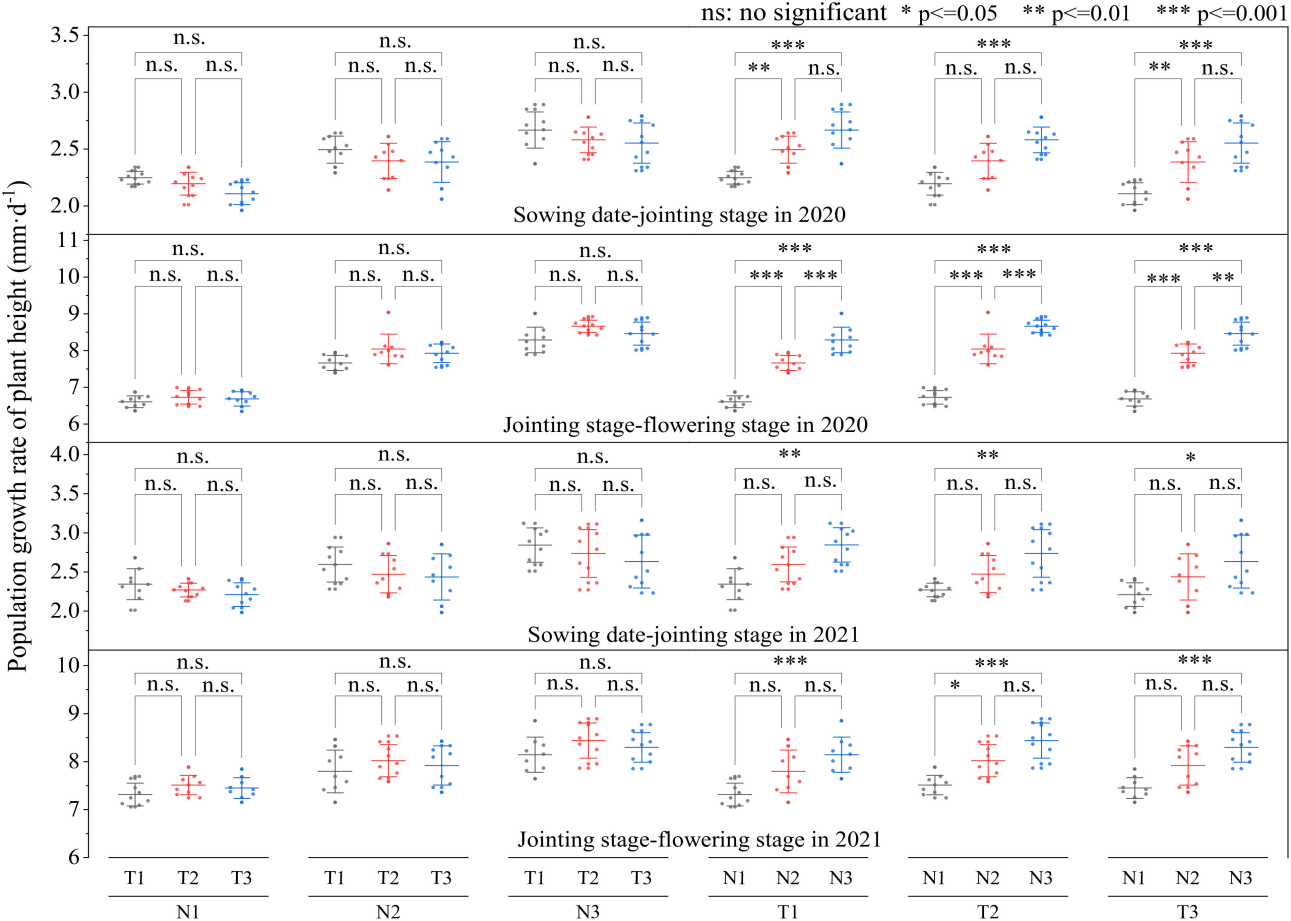
The Effect of optimizing N application on population growth rate of plant height. Values were means ± SD. Different letters indicated significant difference within the same year under the treatments of three N levels and three N topdressing ratios by Duncan test (ANOVA) at the 5% level. Three N fertilization levels: N1, 120 kg ha^-1^; N2, 180 kg ha^-1^; and N3, 240 kg ha^-1^. Three N topdressing ratios: T1, 7:3; T2, 6:4; and T3, 5:5.

At (**Figure 3**) the same N level, the population growth rate of plant height from sowing date to jointing stage in T1 was higher than that in T2 and T3; however, the population growth rate of plant height from jointing stage to flowering stage was consistently T2 > T3 > T1 in 2020 and 2021, and the differences among T1, T2, and T3 were insignificant. With the same N topdressing ratio (T1, T2, or T3), the population growth rate of plant height was consistently N3 > N2 > N1, and the differences between N3 and N1 were significant. The results indicated that N topdressing ratio had no significant effect on the population growth rate of plant height, while increasing 120 kg N ha^-1^ significantly enhanced the population growth rate of plant height.

#### 3.2.2 Dynamic changes of population leaf area

At the same N level (**Table 1**), the leaf area index at the flowering and middle of filling stages and the leaf area growth rate from the jointing stage to flowering stage were consistently T2 > T3 > T1, while the leaf area index at jointing stage and leaf area growth rate from sowing date to jointing stage were consistently T1 > T2 > T3, indicating that increasing the basal N application amount was beneficial to increasing leaf area index and leaf area growth rate of wheat before the jointing stage, and T2 was the most beneficial to improving the leaf area index and leaf area growth rate after the jointing stage. At the same N level, the differences in leaf area index and leaf area growth rate were insignificant between T2 and T3. With the same N topdressing ratio, the leaf area index and leaf area growth rate in N3 were higher than in N2 and significantly higher than that in N1 (except for leaf area growth rate from jointing to flowering stages between N3T1 and N1T1 in 2020). Therefore, increasing the N application amount appropriately with the N topdressing ratio T2 was relatively more helpful for enhancing leaf area index and leaf area growth rate after jointing stage.

**Table 1:** The effect of optimizing N application on dynamic changes of population leaf area.

N fertilization	N topdressing ratio	Leaf area index			Leaf area growth rate (m^2^·ha^-1^d^-1^)	
		Jointing stage	Flowering stage	Middle of filling stage	From sowing date to jointing stage	From jointing stage to flowering stage
2020						
N1	T1	3.25 ± 0.15 b	5.38 ± 0.16 f	3.26 ± 0.17 c	282.32 ± 13.20 b	417.65 ± 36.31 c
	T2	3.22 ± 0.11 b	5.53 ± 0.06 def	3.43 ± 0.13 bc	280.29 ± 9.22 b	452.29 ± 10.06 bc
	T3	3.19 ± 0.14 b	5.43 ± 0.13 ef	3.33 ± 0.20 c	277.68 ± 12.34 b	439.22 ± 21.96 c
N2	T1	3.54 ± 0.22 ab	5.66 ± 0.17 cde	3.49 ± 0.04 bc	307.54 ± 18.70 ab	416.34 ± 61.71 c
	T2	3.48 ± 0.07 ab	5.82 ± 0.14 c	3.65 ± 0.13 ab	302.90 ± 5.79 ab	458.82 ± 18.91 bc
	T3	3.45 ± 0.21 ab	5.73 ± 0.08 cd	3.60 ± 0.10 ab	299.71 ± 18.27 ab	447.06 ± 37.56 c
N3	T1	3.68 ± 0.20 a	6.07 ± 0.14 b	3.67 ± 0.15 ab	319.71 ± 17.27 a	468.63 ± 35.46 bc
	T2	3.63 ± 0.24 a	6.38 ± 0.14 a	3.81 ± 0.09 a	315.36 ± 20.84 a	540.52 ± 57.91 a
	T3	3.61 ± 0.23 a	6.27 ± 0.15 ab	3.77 ± 0.14 a	313.91 ± 19.66 a	521.57 ± 14.14 ab
2021						
N1	T1	3.22 ± 0.15 cde	5.29 ± 0.11 d	3.24 ± 0.21 c	282.16 ± 12.84 cde	398.08 ± 10.71 e
	T2	3.14 ± 0.16 de	5.54 ± 0.11 bcd	3.38 ± 0.16 bc	275.73 ± 13.31 de	461.54 ± 8.81 cd
	T3	3.09 ± 0.15 e	5.47 ± 0.20 cd	3.35 ± 0.29 bc	271.35 ± 12.93 e	456.41 ± 10.94 cd
N2	T1	3.43 ± 0.15 abcd	5.68 ± 0.11 bcd	3.53 ± 0.37 abc	300.88 ± 13.10 abcd	433.33 ± 36.61 d
	T2	3.34 ± 0.19 bcde	5.86 ± 0.17 abc	3.71 ± 0.38 abc	293.27 ± 16.93 bcde	484.62 ± 12.01 bc
	T3	3.30 ± 0.22 bcde	5.78 ± 0.17 abc	3.66 ± 0.34 abc	289.47 ± 19.24 bcde	477.56 ± 10.59 c
N3	T1	3.67 ± 0.13 a	5.97 ± 0.18 ab	3.84 ± 0.34 ab	321.93 ± 10.99 a	442.95 ± 22.78 d
	T2	3.59 ± 0.17 ab	6.24 ± 0.14 a	3.99 ± 0.33 a	315.20 ± 14.61 ab	509.62 ± 13.87 ab
	T3	3.50 ± 0.20 abc	6.19 ± 0.19 a	3.91 ± 0.34 a	307.31 ± 17.11 abc	516.03 ± 7.28 a

values were means ± SD. Means followed by different letters in the same column indicated significant difference within the same year under the treatments of three N levels and three N topdressing ratios by Duncan test (ANOVA) at the 5% level. Three N fertilization levels: N1, 120 kg ha^-1^; N2, 180 kg ha^-1^; and N3, 240 kg ha^-1^. Three N topdressing ratios: T1, 7:3; T2, 6:4; and T3, 5:5.

#### 3.2.3 Dynamic changes of dry matter accumulation of population

At the same N level (**Table 2**), the dry matter weight of the population at the jointing stage were consistently T1 > T2 > T3, while the dry matter weight of the population at the flowering and maturity stages as well as the dry matter accumulation rate from sowing date to flowering stage and from flowering to maturity stage were consistently T2 > T3 > T1, indicating that increasing the basal N application amount was beneficial to increasing the dry matter weight of population before the jointing stage, and the T2 treatment was the most beneficial to improving the dry matter weight and dry matter accumulation rate of the population after the jointing stage. The differences in the dry matter accumulation rate among T1, T2, T3 were insignificant (except between N3T1 and N3T2, N3T3 from sowing date to flowering stage in 2021). With the same N topdressing ratio, the dry matter weight of the population in N2 was significantly higher than that in N1, and in N3 it was significantly higher than that in N2 (except at maturity in 2021), indicating that increasing 60 kg N ha^-1^ significantly enhanced the dry matter weight of population. The dry matter accumulation rate in N3 was higher than that in N2 and significantly higher than that in N1.

**Table 2:** The effect of optimizing N application on the population dynamics of dry matter accumulation.

N fertilization	N topdressing ratio	Dry matter weight of population (kg·ha^-1^)			Dry matter accumulation rate (kg·ha^-1^d^-1^)	
		Jointing stage	Flowering stage	Maturity stage	From sowing date to flowering stage	From flowering to maturity stage
2020						
N1	T1	4001.03 ± 95.26 c	8617.23 ± 120.70 d	13236.13 ± 235.55 e	51.91 ± 1.67 e	131.97 ± 4.47 d
	T2	3943.40 ± 72.56 cd	8763.03 ± 74.89 d	13873.40 ± 274.22 d	52.79 ± 0.45 de	146.01 ± 6.35 bcd
	T3	3839.30 ± 55.06 d	8709.10 ± 62.87 d	13681.87 ± 279.34 de	52.46 ± 2.34 de	142.08 ± 6.23 cd
N2	T1	4219.60 ± 107.59 b	9097.60 ± 65.02 c	14142.47 ± 142.32 cd	54.80 ± 0.39 cde	144.14 ± 2.25 cd
	T2	4163.63 ± 79.47 b	9196.27 ± 57.59 c	14645.13 ± 407.20 bc	55.40 ± 2.22 bcd	155.68 ± 16.53 abc
	T3	4087.70 ± 24.55 bc	9138.30 ± 106.66 c	14468.70 ± 478.71 c	55.05 ± 0.64 cde	152.30 ± 10.78 abc
N3	T1	4457.57 ± 106.72 a	9509.13 ± 46.64 b	15162.37 ± 128.63 ab	57.28 ± 2.21 abc	161.52 ± 2.41 ab
	T2	4419.30 ± 85.06 a	9808.00 ± 55.43 a	15617.80 ± 372.23 a	59.08 ± 2.32 a	165.99 ± 9.39 a
	T3	4365.63 ± 50.20 a	9714.27 ± 82.00 a	15445.27 ± 55.43 a	58.52 ± 0.49 ab	163.74 ± 3.69 a
2021						
N1	T1	3992.57 ± 22.09 ef	8734.67 ± 113.33 e	13903.10 ± 583.78 e	52.62 ± 0.68 e	152.01 ± 13.96 d
	T2	3943.43 ± 69.43 ef	8920.77 ± 108.56 de	14270.97 ± 340.60 de	53.74 ± 0.65 de	157.36 ± 7.26 bcd
	T3	3820.80 ± 71.87 f	8877.37 ± 99.26 e	14165.07 ± 526.57 de	53.48 ± 0.60 e	155.52 ± 12.57 cd
N2	T1	4287.57 ± 149.28 cd	9081.10 ± 152.89 cd	14985.43 ± 797.25 cd	54.71 ± 0.92 cd	173.66 ± 19.10 abc
	T2	4238.53 ± 104.94 cd	9245.00 ± 115.95 c	15336.63 ± 450.55 abc	55.69 ± 0.70 c	179.17 ± 10.41 a
	T3	4113.37 ± 81.83 de	9190.23 ± 144.25 c	15141.43 ± 271.10 bc	55.36 ± 0.87 c	175.04 ± 4.31 abc
N3	T1	4553.80 ± 83.60 a	9612.40 ± 155.45 b	15636.13 ± 189.41 abc	57.91 ± 0.94 b	177.17 ± 2.99 ab
	T2	4484.13 ± 136.17 ab	9871.50 ± 82.57 a	16153.97 ± 258.94 a	59.47 ± 0.50 a	184.78 ± 5.62 a
	T3	4360.47 ± 73.15 bc	9831.70 ± 116.84 a	15950.87 ± 529.15 ab	59.23 ± 0.70 a	179.98 ± 12.70 a

values were means ± SD. Means followed by different letters in the same column indicated significant difference within the same year under the treatments of three N levels and three N topdressing ratios by Duncan test (ANOVA) at the 5% level. Three N fertilization levels: N1, 120 kg ha^-1^; N2, 180 kg ha^-1^; and N3, 240 kg ha^-1^. Three N topdressing ratios: T1, 7:3; T2, 6:4; and T3, 5:5.

#### 3.2.4 Dynamic changes of population light interception rate

**Figure 4:**
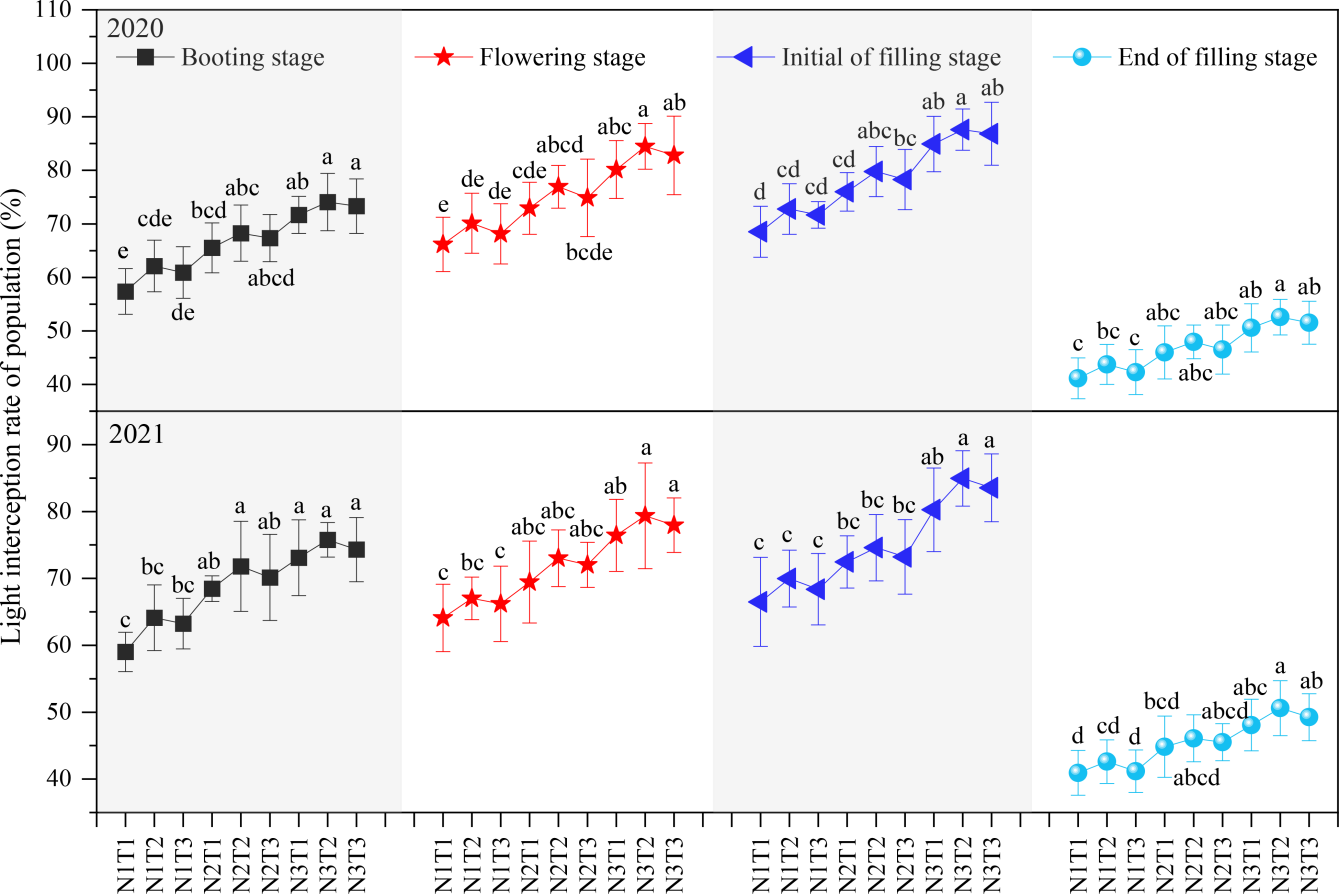
The Effect of optimizing N application on population light interception rate. Values were means ± SD. Different letters indicated significant difference within the same year under the treatments of three N levels and three N topdressing ratios by Duncan test (ANOVA) at the 5% level. Three N fertilization levels: N1, 120 kg ha^-1^; N2, 180 kg ha^-1^; and N3, 240 kg ha^-1^. Three N topdressing ratios: T1, 7:3; T2, 6:4; and T3, 5:5.

At (**Figure 4**) the same N level (N1, N2, or N3), the population light interception rate in T2 was higher than that in T1 and T3 at the same growth stage, but the differences among T1, T2, and T3 were insignificant, indicating that an appropriate N topdressing ratio was beneficial to increasing population light interception rate, but its effect was insignificant. Increasing the N application amount enhanced the population light interception rate; with the same N topdressing ratio, the population light interception rate in N3 was higher than that in N2 and significantly higher than that in N1. The results showed that increasing 120 kg N ha^-1^ significantly enhanced the population light interception rate.

#### 3.2.5 Dynamic changes of population spikelet number

**Figure 5:**
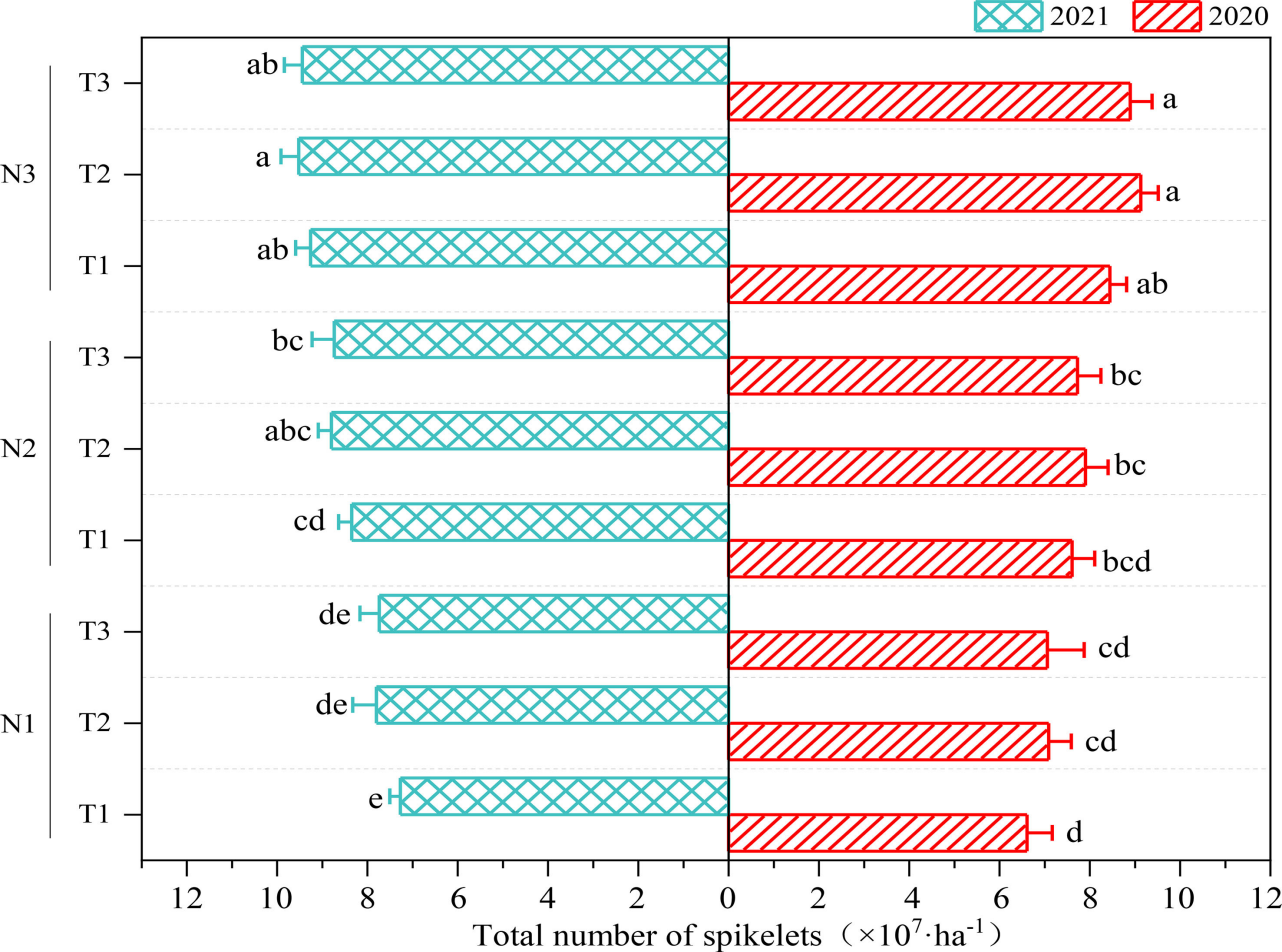
The Effect of optimizing N application on total number of population spikelets. Values were means ± SD. Different letters indicated significant difference within the same year under the treatments of three N levels and three N topdressing ratios by Duncan test (ANOVA) at the 5% level. Three N fertilization level: N1, 120 kg ha^-1^; N2, 180 kg ha^-1^; and N3, 240 kg ha^-1^. Three N topdressing ratios: T1, 7:3; T2, 6:4; and T3, 5:5.

The total (**Figure 5**) number of spikelets in T2 were higher than that in T1 and T3 at the same N level (N1, N2 or N3), but the differences among T1, T2, and T3 were insignificant. With the same N topdressing ratio, the total number of spikelets in N3 were higher than that in N2, and significantly higher than that in N1. We can draw that N topdressing ratio had no significant effect on total number of spikelets, while increasing 120 kg N ha^-1^ significantly enhanced the total number of population spikelets.

### 3.3 Effects of optimizing N application on ear fruiting traits and yield

At the same N level (**Table 3**), the fruiting spikelets per ear, grains per ear, grain weight per ear, fruiting rate per ear, grain filling rate per ear, and yield were consistently T2 > T3 > T1, while the sterile spikelets at the top and bottom and imperfect grains per ear were consistently T1 > T3 > T2, indicating that T2 was not only beneficial to increasing the fruiting spikelets per ear, grains per ear, grain weight per ear, fruiting rate per ear, grain filling rate per ear and yield, but also helpful for decreasing the sterile spikelets at the top and bottom and imperfect grains per ear. Increasing the N application amount enhanced the fruiting spikelets per ear, grains per ear, grain weight per ear, fruiting rate per ear, grain filling rate per ear and yield, while it decreased the sterile spikelets at the top and bottom and imperfect grains per ear. The differences in fruiting spikelets per ear, sterile spikelets at the top and bottom, grains per ear, grain weight per ear, imperfect grains per ear, fruiting rate per ear, and grain filling rate per ear between N3 and N1 were significant. In addition, the yield in N3 was significantly higher than that in N2, and in N2 it was significantly higher than that in N1.

**Table 3:** The effect of optimizing N application on ear fruiting traits (average value of 2020 and 2021) and yield of wheat.

N fertilization	Ratio of basal to topdressing	Fruiting spikelets per ear	Sterile spikelets at the top	Sterile spikelets at the bottom	Grains per ear	Grain weight per ear (g)
N1	T1	12.19 ± 0.57 d	0.60 ± 0.04 a	1.92 ± 0.06 a	27.93 ± 1.04 c	1.03 ± 0.08 e
	T2	13.37 ± 0.45 cd	0.53 ± 0.04 bc	1.79 ± 0.08 abc	28.53 ± 0.77 c	1.08 ± 0.07 de
	T3	13.17 ± 0.89 cd	0.54 ± 0.03 b	1.83 ± 0.08 ab	28.07 ± 0.60 c	1.06 ± 0.05 e
N2	T1	13.86 ± 0.90 bc	0.50 ± 0.03 bcd	1.73 ± 0.08 bc	30.71 ± 1.25 b	1.19 ± 0.05 cd
	T2	14.48 ± 0.52 bc	0.46 ± 0.03 def	1.66 ± 0.09 cde	31.89 ± 1.24 ab	1.26 ± 0.05 abc
	T3	14.10 ± 1.34 bc	0.48 ± 0.04 cde	1.69 ± 0.07 bcd	31.57 ± 2.31 ab	1.24 ± 0.07 bc
N3	T1	15.24 ± 0.23 ab	0.43 ± 0.04 efg	1.58 ± 0.09 def	32.22 ± 1.43 ab	1.30 ± 0.05 abc
	T2	16.59 ± 0.90 a	0.39 ± 0.03 g	1.51 ± 0.06 f	33.11 ± 0.76 a	1.36 ± 0.05 a
	T3	16.18 ± 0.69 a	0.40 ± 0.02 fg	1.53 ± 0.05 ef	32.75 ± 0.77 ab	1.34 ± 0.08 ab
		Imperfect grains per ear	Fruiting rate per ear (%)	Grain filling rate per ear (mg·d^-1^)	Yield in 2020 (kg·ha^-1^)	Yield in 2021 (kg·ha^-1^)
N1	T1	2.45 ± 0.12 a	86.41 ± 0.80 e	36.79 ± 2.14 d	4300.73 ± 50.86 e	4386.27 ± 43.52 d
	T2	2.26 ± 0.12 b	88.38 ± 1.86 cde	38.57 ± 2.74 d	4378.07 ± 20.91 e	4427.80 ± 60.60 d
	T3	2.28 ± 0.09 ab	87.72 ± 1.16 de	37.98 ± 2.58 d	4322.60 ± 38.07 e	4404.77 ± 86.37 d
N2	T1	2.03 ± 0.08 c	89.50 ± 0.96 bcd	42.38 ± 2.51 c	5444.83 ± 46.90 d	5423.60 ± 54.85 c
	T2	1.88 ± 0.09 cd	90.77 ± 1.40 ab	44.88 ± 3.77 bc	5566.50 ± 49.19 c	5548.87 ± 69.08 b
	T3	1.92 ± 0.10 c	90.15 ± 1.40 bc	44.17 ± 2.78 bc	5516.57 ± 71.00 cd	5493.80 ± 78.71 bc
N3	T1	1.74 ± 0.12 de	91.60 ± 1.30 ab	46.31 ± 2.03 ab	5814.80 ± 100.25 b	5894.17 ± 86.70 a
	T2	1.60 ± 0.08 e	92.99 ± 1.24 a	48.69 ± 1.95 a	5995.53 ± 111.67 a	5963.50 ± 72.98 a
	T3	1.64 ± 0.08 e	92.59 ± 1.42 a	47.86 ± 2.07 ab	5917.73 ± 53.19 ab	5930.30 ± 80.24 a

values were means ± SD. Means followed by different letters in the same column indicated significant difference within the same year under the treatments of three N levels and three N topdressing ratios by Duncan test (ANOVA) at the 5% level. Three N fertilization levels: N1, 120 kg ha^-1^; N2, 180 kg ha^-1^; and N3, 240 kg ha^-1^. Three N topdressing ratios: T1, 7:3; T2, 6:4; and T3, 5:5.

### 3.4 Correlation analysis

**Figure 6:**
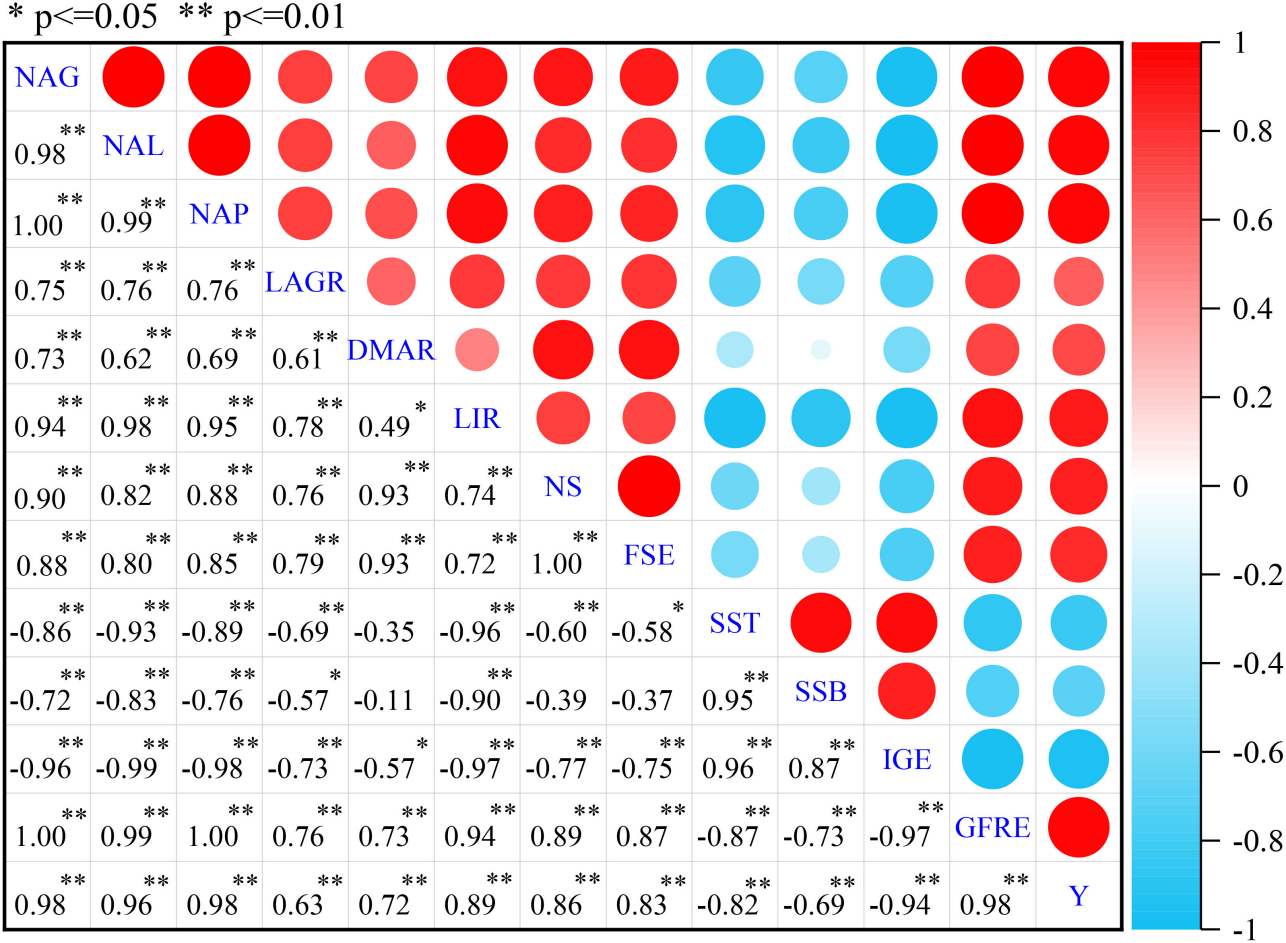
Correlation analyses among NAG (N accumulation amount in grain), NAL (N accumulation amount in leaf), NAP (N accumulation amount in plant), LAGR (leaf area growth rate) and DMAR (dry matter accumulation rate), LIR (light interception rate of population), NS(number of population spikelets), FSE (fruiting spikelets per ear), SST (sterile spikelets at the top), SSB (sterile spikelets at the bottom), IGE (imperfect grains per ear), GFRE (grain filling rate per ear), and Y (yield). *Correlation is significant (p<0.05); **Correlation is markedly significant (p<0.01).

As shown in **Figure 6**, the NAG (N accumulation amount in grain), NAL (N accumulation amount in leaf), NAP (N accumulation amount in plant), LAGR (leaf area growth rate), DMAR (dry matter accumulation rate), LIR (light interception rate of population), NS(number of population spikelets), FSE (fruiting spikelets per ear), GFRE (grain filling rate per ear), and Y (yield) were all significantly (p<0.05) positively correlated with each other, indicating that there were positive (significant or markedly significant) mutual promotion interactions among each factor. The SST (sterile spikelets at the top), SSB (sterile spikelets at the bottom), and IGE (imperfect grains per ear) were all significantly negatively correlated with NAG, NAL, NAP, LAGR, DMAR, LIR, NS, FSE, GFRE, and Y (except for the relationships between SSB with DMAR, NS, FSE, and between SST with DMAR). The relationships between SST, SSB, and IGE were significant, indicating that the increase of sterile spikelets would lead to an obvious enhancement of imperfect grains per ear. Therefore, to increase N accumulation amount in grain, leaf, and plant, and the rate of leaf area growth, dry matter accumulation and population light interception by optimizing N application amount and topdressing ratio have great significance on reducing the number of sterile spikelets and imperfect grains and increasing the number of fruiting spikelets per ear, grain filling rate, and yield.

## 4 Discussion

Precise N fertilization management is important in improving plant N uptake and N use efficiency (NUE), while increasing the N fertilization level is unlikely to be effective in enhancing potential N benefits and NUE (Wu et al., 2019). Better management and appropriate use of N fertilizers are a convenient and effective way to improve crop productivity and N use efficiency with minimum N loss (Yin et al., 2019). Therefore, optimizing N application strategies should consider adopting an appropriate N fertilization rate and splitting the fertilizer dose into different fertilization or topdressing application times based on crop requirements so as to enhance N use efficiency while decrease N inputs. Liu et al. (2019) indicated that in the split application of N, where some N was applied before sowing and some N was applied at later growth periods, N concentration in the grain, total N accumulation at harvest, and N use efficiency increased. Sun et al. (2020) found that compared with one-time basal fertilization, optimized N topdressing ratios (especially the 4:3:3 and 5:3:2 ratios) significantly increased N use efficiency and aboveground N accumulation. In this study, we found that T3 was more beneficial to increasing total N accumulation in the stem plus petiole compared with T1 and T2, while T1 was more beneficial to enhancing N use efficiency for grain and biomass production compared with T2 and T3, and the effect of the N topdressing ratio on N accumulation amount in leaf, grain, plant, and partial factor productivity of applied N were consistently T2 > T3 > T1. In addition, we also proved that increasing 60 kg N ha^-1^ significantly enhanced N accumulation amount in the stem plus petiole, leaf, grain, and plant, while partial factor productivity of applied N (within the range of N application in this experiment) significantly decreased, and a similar conclusion was also drawn by Zhao and Si (2015). Di Paolo and Rinaldi (2008) found that N use efficiency (NUE) decreased gradually as the N level increased from 0 to 300 kg ha^-1^, while excessive N application did not enhance N uptake. Jia et al. (2011) also reported that the optimal ratio of basal-N to topdress-N could lead to higher N accumulation in wheat, higher N recovery efficiency, NUE, and decreased loss of fertilizer-N. Optimizing the N application rate and N topdressing ratio could have a significant impact on plant N accumulation and NUE, which is mainly due to the fact that (i) as the rate of N application relative to plant N requirement increases, the N use efficiency normally decreases; and (ii) delaying N application until the later growth stage or applying N with a suitable topdressing ratio is an effective strategy for improving the synchrony among N supply, soil N availability, and crop N demand (Gu et al., 2019; Liu et al., 2019).

Plant height, leaf area, dry matter weight and spikelet number of population are important agronomic traits of cereal crops that not only determine plant architecture but also contribute significantly to grain yield. For instance, Machado et al. (2002) indicated that plant height explained 61% of the variation in the grain yield of corn. Viña et al. (2011) reported that leaf area was an important parameter controlling many biological and physical processes of the crop, including the interception of light and water, autotrophic respiration, dry matter accumulation, and carbon and nutrient cycles. Therefore, it is of great significance to study and reveal the effects of N application amount and topdressing ratio on agronomic characters, especially on the growth rate of plant height, leaf area, dry matter weight, and spikelet number of population. Chen et al. (2016) reported that maize plants with 220 kg N ha^-1^ had a higher dry matter accumulation, green leaf number and leaf area index than plants with 55 kg N ha^-1^. Ye et al. (2019) indicated that increasing tiller N could help promote tillering during the early growth stage, and increasing panicle N could help increase the number of differentiated spikelets (prevent differentiated spikelets from degeneration), as well as enhance dry matter accumulation and the percentage of filled grains. In this study we found that increasing basal N application amount(T1)was beneficial to increasing the population growth rate of plant height, leaf area index, leaf area growth rate, and dry matter weight (from sowing date to jointing stage), while after the jointing stage, T2 showed more advantages in increasing the population growth rate of plant height, leaf area index, leaf area growth rate, dry matter weight, dry matter accumulation rate, and spikelets of population. In addition, we also found that the dry matter weight of population was significantly enhanced by increasing 60 kg N ha^-1^, and the population growth rate of plant height, leaf area index, leaf area growth rate, dry matter accumulation rate, and spikelets of population were significantly enhanced by increasing 120 kg N ha^-1^. The effect of increasing 60 kg N ha^-1^ on improving the above-mentioned indexes were more obvious than that of N topdressing ratio. Some studies (Tian et al., 2017; Ye et al., 2019; Xu et al., 2021) also proved that the population dynamics or population structure of crops were significantly influenced by N fertilization level and N topdressing ratio, for which the results presented in this paper provided further evidence. Therefore, we can obtain ideal population dynamics (structure) of crops by choosing a suitable combination of N application rate and N topdressing ratio.

Crop yield formation is closely related to the efficient use of radiation resources, and enhancing crop ability to capture radiation resources is an effective strategy for increasing crop productivity. Xue et al. (2015) indicated that the amount of light intercepted by the crop population reflected the physiological processes that occur in the population, and that the interception of light by the crop population was complicated and was affected by some agricultural measures. Zhao et al. (2022) reported that a fraction of intercepted photosynthetic active radiation (FIPAR) could be regulated through an N application method, which was lower in the N limiting treatments than in the N non-limiting treatments. In this study we found that an appropriate N topdressing ratio (T2) was conductive to increasing the light interception rate of population, but the effect was insignificant. In addition, increasing 120 kg N ha^-1^ significantly enhanced the light interception rate of population within the amount of N application in this experiment. These results were similar to those of Tsujimoto et al. (2017) who also proved that suitable N application methods could obviously improve crop population development and increase both radiation use efficiency and light interception. N application amount and topdressing ratio can obviously affect the light interception rate of population mainly due to plant density and leaf area expansion not being closely related to the intercepted PAR within the population but also significantly affected by N supply status and period (Tao et al., 2018; Zhao et al., 2022).

Agronomically optimizing the timing and rates of N fertilizer application can enhance crop yield and coordinate yield component parameters. Liu et al. (2019) indicated that by applying N fertilizer with three splits and delaying topdressing fertilization until the booting stage of winter wheat, the total grain yield and spike number, kernel number per spike, and 1000-kernel weight could increase. In some similar studies, it is also reported that by dividing N fertilizer application into basal and topdressing applications, the source–sink relationship could be regulated, further improving yield component parameters and increasing crop yields (Ercoli et al., 2013; Sui et al., 2013; Lyu et al., 2021). In this study, we found that N topdressing ratio T2 was not only beneficial to increasing fruiting spikelets per ear, grains per ear, grain weight per ear, fruiting rate per ear, grain filling rate per ear, and yield but was also helpful for decreasing sterile spikelets at the top and bottom and imperfect grains per ear. In addition, enhancing the N application rate from 120 kg ha^-1^ to 180 kg ha^-1^ or from 180 kg ha^-1^ to 240 kg ha^-1^ significantly increased yield and obviously improved the above-mentioned yield component parameters. A suitable N application rate and N topdressing ratio can obviously increase crop yield and improve yield component parameters mainly because (i) compared with one-time basal fertilization, aboveground N accumulation and NUE of crops can be significantly enhanced with an optimized N application rate and N topdressing ratio (Liu et al., 2019; Yin et al., 2019); (ii) N supply is better synchronized with crop N demand (Shi et al., 2012; Gu et al., 2019); (iii) physiological characteristics, population dynamics, and structures closely related to yield formation are effectively improved.

## 5 Conclusions

The effects of N topdressing ratio on increasing N accumulation amounts in leaf, grain, and plant and partial factor productivity of applied N were consistently 6:4 > 5:5 > 7:3, but N topdressing ratio 7:3 was more conducive to improving N use efficiency for grain and biomass production, and increasing 60 kg N ha^-1^ significantly enhanced the N accumulation amount in the stem plus petiole, leaf, grain, and plant. After the jointing stage, a N topdressing ratio 6:4 was more beneficial to enhancing the population growth rate of plant height, leaf area index, leaf area growth rate, dry matter weight, dry matter accumulation rate, light interception rate, and the total number of population spikelets; meanwhile, the above-mentioned indexes of population could be significantly enhanced by increasing 120 kg N ha^-1^. N3T2 was more conducive to improving yield and yield component parameters, and increasing 60 kg N ha^-1^ significantly enhanced yield. Therefore, we suggested that a moderate N application rate N3 with a suitable N topdressing ratio might be an environmentally friendly mode for wheat cropping for high yield (T2) and N use efficiency (T1).

## Data availability statement

The original contributions presented in the study are included in the article/supplementary material. Further inquiries can be directed to the corresponding authors.

## Author contributions

XZ conducted the field experiments and wrote the manuscript. SD, YX, and CC provided advice on experimental implementation. HC and YQ prepared the materials for the experiment. YQ and SD conceived and supervised the field experiments and revised manuscript. YX, CC, and HC participated data collection and data analysis. All authors contributed to the article and approved the submitted version.
